# Psychosocial stressors predict lower cardiovascular disease risk among Mexican-American adults living in a high-risk community: Findings from the Texas City Stress and Health Study

**DOI:** 10.1371/journal.pone.0257940

**Published:** 2021-10-07

**Authors:** Maryam Hussain, Jennifer L. Howell, M. Kristen Peek, Raymond P. Stowe, Matthew J. Zawadzki

**Affiliations:** 1 Department of Psychological Sciences, University of California-Merced, Merced, California, United States of America; 2 Department of Preventive Medicine and Population Health, University of Texas Medical Branch-Galveston, Galveston, Texas, United States of America; 3 Microgen Laboratories, La Marque, Texas, United States of America; University of Colorado Denver, UNITED STATES

## Abstract

The objective of this study was to examine the link between systemic and general psychosocial stress and cardiovascular disease (CVD) risk in a group of U.S. Latinos as a function of acculturation and education within the blended guiding conceptual framework of the biopsychosocial model of the stress process plus the reserve capacity model. We analyzed data from self-identifying Mexican-origin adults (*n* = 396, 56.9% female, *M*_age_ = 58.2 years, 55.5% < 12 years of education, 79% U.S.-born) from the Texas City Stress and Health Study. We used established measures of perceived stress (general stress), neighborhood stress and discrimination (systemic stress) to capture psychosocial stress, our primary predictor. We used the atherosclerotic CVD calculator to assess 10-year CVD risk, our primary outcome. This calculator uses demographics, cholesterol, blood pressure, and history of hypertension, smoking, and diabetes to compute CVD risk in the next 10 years. We also created an acculturation index using English-language use, childhood interaction, and preservation of cultural values. Participants reported years of education. Contrary to expectations, findings showed that higher levels of all three forms of psychosocial stress, perceived stress, neighborhood stress, and perceived discrimination, predicted lower 10-year CVD risk. Acculturation and education did not moderate the effects of psychosocial stress on 10-year CVD risk. Contextualized within the biopsychosocial and reserve capacity framework, we interpret our findings such that participants who accurately reported their stressors may have turned to their social networks to handle the stress, thereby reducing their risk for CVD. We highlight the importance of examining strengths within the sociocultural environment when considering cardiovascular inequities among Latinos.

## Introduction

Cardiovascular disease (CVD) is the leading cause of death in the U.S., with almost one-quarter of deaths result from CVD [1]. CVD has a variety of causes, including medical conditions and lifestyle choices [2]. Relevant to the present endeavor, the Biopsychosocial Model of the stress process highlights the link between environmental demands (i.e., psychological and social stressors) and the physiological and/or behavioral responses to those stressors [3, 4], which predict increased risk for chronic diseases, such as CVD [5–7]. Unfortunately, most studies examining the link between psychosocial stress and CVD do not include multidimensional forms of stress [8, 9] and focus on non-Hispanic/Latino/a/x populations [10]. Keeping in tradition with the Spanish language we henceforth use Latino as a gender-neutral term to refer to those of Hispanic/Latino/a/x origin. U.S. Latinos comprise more than half the of the total U.S. population growth [11], and CVD is the leading cause of death amongst Latinos [12, 13]. However, correlates of CVD risk are not well understood amongst this population [14–16].

Consistently, the American Heart Association encourages examining social and cultural correlates of CVD among Latinos [10]. One such proposed model to better understand the modifying correlates of the relationship between stress and CVD risk among U.S. Latinos is the Reserve Capacity Model, which considers the sociocultural resource factors underlying health disparities related to social and economic disadvantages amongst this population [14]. Here, we propose that multiple types of stressors influence CVD among Latino adult populations, and that their sociocultural resources may moderate this relationship. Specifically, we examine the link between systemic and general psychosocial stress and CVD risk in a group of U.S. Latinos as a function of acculturation and education. We use the blended biopsychosocial stress and reserve capacity models conceptual framework [14] to guide our understanding of these associations. Additionally, we focus on whether different types of stress (i.e., systemic versus general) might relate differently to CVD as a function of these two sociocultural factors.

### Systemic and general stressors

U.S. Latino adults experience a variety of traumatic and chronic stressors in conjunction with other more general forms of stressors [7]. Indeed, a survey conducted by the American Psychological Association [17] indicated that compared to other ethnic or racial groups, Latinos reported experiencing the highest levels of stress. In the present research we focus on two types of stressors that Latino adults experience: systemic stressors and general stressors. Systemic stressors, often constrained specifically to certain social group experiences, reflect systemic inequities and disparities within society [18]. Furthermore, systemic stressors underscore the link between lived sociocultural experience and health risk and status [14]. Within the context of this research, we focus on two forms of systemic stressors experienced by U.S. Latino adults: neighborhood stress and ethnic discrimination.

Neighborhood stress is stress brought on by living in unsafe and low-resource neighborhoods [19]. U.S. Latino adults increasingly live in high-stress neighborhoods where infrastructure is underdeveloped or failing (e.g., broken roads, few public transportation options), there are high rates of poverty and crime, and residents lack easy access to resources like medical facilities and healthy grocery options [20–22]. Exposure to these ongoing neighborhood problems can influence a direct physiologic response among Latino adults, such as increased hypertension [23], prevalence of obesity or higher body mass index [24], or inhibit healthy behaviors, such as physical activity [25], which can contribute to increased CVD risk.

Another systemic stressor that we examine is perceived ethnic discrimination, or the appraisal of unfair treatment based on one’s ethnic background [26]. Seminal work in the area of racism (often used interchangeably for ethnic discrimination) within the context of the biopsychosocial model of stress suggests that the perception of an event as racist adversely influences physiological responses [27], such as a hypertension [28] or cardiac events (e.g., myocardial infarction) [29]. Indeed, mounting evidence indicates Latinos continue to experience higher and more-frequent rates of perceived ethnic discrimination than before and that these experiences serve as a chronic and systemic stressor [30–33], putting them at increased CVD risk. However, most of the empirical work on the relationship between cardiovascular health and ethnic discrimination is among African American/Black adults, with limited research focusing on U.S. Latino adults [34].

General stressors, often defined as random stress, tend to occur with similar probability across all social groups [35]. Most common within the biopsychosocial model of stress is perceived stress [23], which is one’s appraisal of the inability to cope with life’s demands, overall [36, 37]. Whereas systemic stressors link the lived *sociocultural* experience to health risk and status, general stressors link the lived *individual* experience to health risk and status [35]. The appraisal of one’s coping abilities can influence physiological and behavioral changes [38], such as elevated body mass index and increased blood pressure [39]. A variety of factors contribute to high levels of general stress among Latino adults [40], including the state of the world and nightly news and the well-being/safety of their families [41, 42], which taken together can impact CVD risk.

However, general stress rarely is considered when examining CVD risk as a function of multidimensional stressors among Latino adults. Empirical research has largely focused on stressors that may be more culturally-nuanced (e.g., acculturative stress, minority stress, ethnic when considering the stress-CVD risk link [43]). We could only find one empirical article that highlighted the effects of multidimensional stressors, specifically including both systemic and general stress, on cardiovascular-related health outcomes among a cohort of Latino adults [44]. Given that Latino adults likely experience both systemic and general stressors in their everyday life [23], we first aimed to examine the link between these two types of psychosocial stressors and CVD risk among a group of U.S. Latino adults using a biopsychosocial model of stress framework.

### Sociocultural modifiers of stress

In addition to having multidimensional experiences with stress, Latino adults also have access to a variety of sociocultural resources that can potentially reduce the harmful effects of stress on health. Within this vein, we draw upon the blended reserve capacity model and biopsychosocial stress model proposed by Gallo and colleagues [14] to understand how relevant sociocultural resources might moderate the relationship between stress and CVD risk among Latino adults. This blended model states that there are social and culturally driven processes that serve as possible protective or risk enhancing for Latino health. That is, the adverse effects of stress on CVD risk may be enhanced by fewer or lower resources to manage the demands created by stress. Conversely, other sociocultural resources may reduce the negative association between stress and CVD risk, as those resources are replenishing and allow for appropriate management of the demands of stress (i.e., the Reserve Capacity) [14, 45]. Furthermore, this blended model asserts that there are synergistic or interaction effects of the sociocultural resources with stress that explain Latino health. We draw upon the reserve capacity model [45] to focus on two potentially relevant sociocultural resources that might interactively moderate the relationship between stress and CVD risk in Latino adults within the context of sociocultural resources: acculturation and education.

First, based on the blended reserve capacity and biopsychosocial stress models, we suspect that acculturation, or the personal adoption of the U.S. mainstream culture, might enhance the relationship between stress and CVD risk. Research links acculturation to more awareness of stressors, such as pervasive societal inequities like ethnic discrimination, and the development of coping mechanisms in response to stress [46–48], such as engaging in poor health behaviors. The magnitude of effect of coping responses to stress may depend on the availability of cultural resources [27], such as social support from family and friends. Indeed, extant literature provides support for poor health behaviors (e.g., poorer diet, less exercise) in response to stress among more acculturated Latinos [49–51]. However, as indicated by the blended reserve capacity and biopsychosocial framework, acculturation is often operationalized by proxy indicators, such as place of birth or nativity [14]. Therefore, we take the multidimensional approach, taking into consideration values and behaviors, to examine how acculturation may enhance the effects of stress on CVD risk.

Second, we suspect that low education, or socioeconomic disadvantage, might enhance the effects of stress on CVD risk. The burden of CVD among U.S. Latinos may be particularly carried by those who are low socioeconomic status (SES) [52]. Furthermore, U.S. Latinos who have lower educational attainment—the most widely used indicator of SES [53]—may also have access to fewer resources to mitigate the effects of stress. Among ethnicities that have a minority status, higher educational attainment is linked to accessibility to coping resources [54, 55] and better health care [56]. Thus, lower education may enhance the effects of stress on CVD risk. Furthermore, taken together, we assert that the interaction of acculturation and education, two prominent sociocultural resources as indicated by Gallo and colleagues [14], is relevant to understanding CVD risk within the biopsychosocial model of stress among Latino adults.

### The current study

This study first tested the link between psychological stress and CVD risk among a group of Latino adults. We specifically examined the link between 10-year CVD risk as a function of both systemic and general stressors: neighborhood stressors and perceived ethnic discrimination, and perceived stress. We hypothesized based on extant literature that higher psychosocial stress, in all forms, would be associated with higher CVD risk. We also explored how interactions between acculturation and education moderated the psychosocial stress-CVD relationship. We hypothesized that the adverse effects of psychosocial stress on CVD would be exacerbated for people who are more acculturated and have less education. These hypotheses were guided by the blended conceptual framework of the biopsychosocial stress and reserve capacity models.

## Materials and method

### Data

We analyzed data from the Texas City Stress and Health Study collected between 2004 and 2006 in Texas City, Texas, a small urban setting in close proximity to a large complex of petrochemical plants [57–59]. This area is considered an environmental risk-scape [60] due to its history of large-scale oil refinery explosions (see 1947 Texas City disaster), one of which occurred during the time of data collection (see 2005 Texas City Refinery explosion). The dataset was designed to study sociobiological stress and health in a representative sample of individuals from multiple ethnic/race groups (*N* = 2706). The study utilized U.S. Census Bureau Current Population Survey methods for listing, enumeration, and interviewing—yielding a response rate of 80 percent. The institutional review board at University of Texas Medical Branch approved the study protocol, and informed consent was obtained from all participants.

### Participants

Although adults of multiethnic backgrounds participated in the study, the current investigation is limited to those individuals aged ≥ 39.5 years at the time of interview who self-identified as Hispanic/Latino, which yielded a sample size of *n* = 396. The age cutoff was used because age ~40 years is when the risk of developing a CVD becomes meaningful [61], thus allowing us to calculate a 10-year CVD risk score. Our sample was 56.9% female, *M*_age_ = 58.2 years, 57% has less than 12 years of formal education, 79.0% were U.S.-born of Mexican-origin and 20.2% were Mexican-born (0.8% did not provide a response).

### Measures

#### Predictor: Neighborhood stress

We measured neighborhood stress using the Perceived Neighborhood Scale (PNS) [62]. Specifically, participants indicated their perception of their own social embeddedness, sense of community, satisfaction with their neighborhood, and crime on 34 items using a scale ranging from “1/very likely or strongly agree” to “5/very unlikely or strongly disagree” (e.g., “I would move out of my neighborhood if I could.”). The original researchers scored the PNS by reversing negatively oriented items and summing across the items (*M* = 105.85, SD = 18.87, α = .91). Higher scores indicated a less stressful neighborhood, thus, we reverse-ranked the participant scores to achieve a measure of neighborhood stress. As such, the highest score (i.e., 139) received the lowest rank (i.e., 1), with higher ranks indicating greater neighborhood stress (*M* = 31.94, *SD* = 18.20).

#### Predictor: Perceived ethnic discrimination

We measured perceived ethnic discrimination with three items adapted from Finch and colleagues [30]. Specifically, participants indicated whether they felt unaccepted by others due to their Hispanic culture (1 = yes, 2 = no), how often they felt treated unfairly due to being Hispanic (1 = always, 4 = never), and how often they had seen friends treated unfairly due to being Hispanic (1 = always, 4 = never). We reverse coded each single-item response, z-transformed them, and then averaged to create a perceived discrimination composite score, with higher values representing more experiences of discrimination (α = .72).

#### Predictor: Perceived stress

We used the Perceived Stress Scale (PSS) [63] to examine participants’ subjective appraisal of their stress. Specifically, participants responded to 10-items where they indicated the frequency of their thoughts and feelings regarding the frequency with which they felt (un)able to cope with stressors over the past month using a scale ranging from “0/never” to 4/very often” (e.g., “In the last month, how often have you felt nervous and ‘stressed’?”). The original investigators of the Texas City data obtained PSS scores by reversing responses to the four positively stated items (e.g., “In the last month, how often have you felt confident about your ability to handles your personal problems?”) and then summing across the 10 items. Higher scores indicate greater perceived stress (α = .85).

#### Outcome: 10-year CVD risk

We used the atherosclerotic CVD algorithm published in the ACC/AHA Guideline on the Assessment of Cardiovascular Risk [61] to compute CVD risk [64]. This validated calculator establishes risk as a quantitative estimation of absolute risk based upon data from representative population samples [65–67]. The algorithm incorporates age, sex, race, total cholesterol (mg/dL), high-density lipoprotein (HDL) cholesterol (mg/dL), systolic and diastolic blood pressure (mmHg), treatment for high blood pressure, cigarette smoking, and diagnosis of diabetes. The calculator uses three race categories: “White”, “African American”, and “Other”. All participants in our sample identified both their ethnicity and race as “Hispanic”; thus, we categorized all participants as ‘Other’ in the calculator. We used blood samples to measure total and HDL cholesterol, and averaged across two blood pressure measurements to detect systolic and diastolic blood pressure. To measure biomarkers, a trained phlebotomist drew blood into EDTA tubes in a centrally located clinic or in the subject’s home in the morning between 0800 and 1100 after fasting. Blood samples were centrifuged to obtain plasma, which were stored in 1-mL aliquots at −70°C until testing. All specimens were batch analyzed and read blind-coded. Detailed information on the blood collection protocol is available elsewhere [68, 69]. Participants self-reported all other variables.

Although the primary purpose of the CVD calculator is for implementation of appropriate therapeutics (e.g., cholesterol treatment, statin therapy) for adults, it seems to produce mixed therapeutic guidelines for Hispanic adults [70, 71]. However, we used it solely to identify population risk and not for providing recommendations about or implementation of therapeutics. On average, the sample had a 16.95% risk, or intermediate risk, of developing a CVD in the next 10 years. According to the risk calculator, less than 5% is considered low-risk, 5% to 7.4% is considered borderline risk, 7.5% to 19.9% is considered intermediate risk, and 20% or more is considered high risk.

#### Moderating factor: Acculturation

Hazuda and colleagues [72] encourage using an index of acculturation that measures English-language use, childhood interaction, and preservation of culture when studying adults of Mexican-origin. In line with that prior work, and other published work from the Texas City Stress and Health Study, we created an index of acculturation by combining responses on English-language use, childhood interaction, and preserving Mexican culture [59, 68]. The English-language use measure comprised 6 items asking about primary language spoken as a child and adult (α = .94). A sample item is “What language was spoken in your home when you were a child?” with responses ranging from 1 (*only Spanish*) to 5 (*only English*). The childhood interaction measure comprised 3 items that asked about the ethnicity of people with whom respondents had associated when they were children (α = .90). A sample item is “When you were growing up, were your neighbors…” with responses ranging from 1 (*mostly Spanish/Hispanic/Latino)* to 3 *(mostly Anglo)*. The preserving Mexican culture measure comprised 3 items reflecting the importance of children knowing the history and customs of Mexico (α = .75). A sample item is “How important do you feel it is for children to follow Mexican customs and ways of life?” with responses ranging from 1 (*very important*) to 4 (*not important at all*). We z-transformed and then averaged items from all 3 measures to create a composite index of acculturation, with higher scores reflecting greater acculturation. A confirmatory factor analysis indicated an acceptable model fit for a single-factor model, χ^2^ = 111.12, *df* = 2, p < .001; CFI = .93, RMSEA = .06.

#### Moderating factor: Education

Consistent with other work using education as a proxy for SES [39], we assessed participants level of education. An advantage of using education as an objective measure of SES for adults is that reverse causation is reduced. That is, educational attainment typically occurs before detrimental health arises [73]. Participants indicated how many years of school they had completed (55% less than 12 years).

#### Statistical analyses

We computed descriptive statistics and correlations for age, sex, education, psychosocial factors, CVD risk factors and 10-year CVD risk. We then examined the moderating role of acculturation and education on the main effects of psychological stress using hierarchical linear regression models. All continuous predictor and moderating variables were mean-centered prior to computing interaction terms. Furthermore, all psychosocial stress variables were z-transformed to allow for easier comparison of the different stressors within the same model. Because demographics (age, sex, and ethnicity) are included in 10-Year CVD risk, we did not adjust for them in the model. However, nativity correlated with treatment for blood pressure—a key risk factor for CVD—and therefore, was included as a covariate. The hierarchical models were set so that first the systemic stressors were entered (neighborhood stress and perceived ethnic discrimination); second, general stress (i.e., perceived stress) was entered; third, the sociocultural modifiers were entered (acculturation and education). Subsequent steps (fourth and fifth) included the various two-way and three-way interaction effects of the psychological stressors, acculturation, and education. Finally, the sixth step included adjustment for nativity. All analyses were conducted in SPSS v. 25 (Armonk, NY).

## Results and discussion

Table 1 presents descriptive statistics for all variables.

**Table 1:** Participant demographic, psychological stressors, sociocultural, and cardiovascular risk factor characteristics (*n* = 396).

	*M* (SD) or %	range
***Demographic Variables***		
Age (years)	57.26 (11.41)	39.54–79.32
Sex (% female)	56.8	
U.S. Born (%)	79.0	
***Psychological Stressors- Predictors***		
Perceived Stress	12.33 (7.71)	0–40
Neighborhood Stress	31.94 (18.20)	1–88
Discrimination^z^	0.01 (0.82)	-0.65–3.07
***Sociocultural Variables- Moderators***		
Education	10.02 (3.67)	0–20
Preserving Mexican Culture	5.32 (2.59)	3–15
English Language Use	18.13 (5.73)	6–28
Childhood Interaction	5.07 (1.81)	3–9
Acculturation^z^	0.00 (.74)	-1.39–2.16
***CVD-Related Variables-Outcome***		
Systolic blood pressure, mmHg	127.13 (19.52)	74–240
Diastolic blood pressure, mmHg	76.51 (11.21)	40–130
Total Cholesterol, mg/dL	209.06 (41.98)	102–357
HDL cholesterol, mg/dL	50.14 (14.76)	23–119
HbA1c%	6.56 (1.84)	1–14
BP Treatment (% yes)	52.1	
Diabetic (% yes)	65.2	
Smoker (% at least 1 year)	45.3	
CVD Risk (% in 10 years)	16.95(14.26)	0–72
Low CVD Risk (<5%)	22.5	
Borderline CVD Risk (5%-7.4%)	11.1	
Intermediate CVD Risk (7.5%-19.9%)	29.8	
High CVD Risk (≥20%)	36.6	

Note.

^z^ denotes standardized index variable.

Table 2 presents correlations of all variables. Of note, higher systolic blood pressure and hemoglobin A1c (HbA1c %), lower HDL, being diagnosed with diabetes, and not being treated for high blood pressure all significantly correlated with higher 10-year CVD risk. Interestingly, smoking for at least one year within the lifetime correlated with lower 10-year CVD risk. Diastolic blood pressure and total cholesterol did not correlate with 10-year CVD risk. Surprisingly, higher levels in all types of psychological stressors, perceived neighborhood stress, *r*(394) = -.14, *p* = .017, perceived discrimination, *r*(394) = -.13, *p* = .011, and perceived stress, *r*(394) = -.18, *p* = .001, were all associated with lower 10-year CVD risk.

**Table 2:** Correlations of all study variables (*n* = 393).

	1.	2.	3.	4.	5.	6.	7.	8.	9.	10.	11.	12.	13.	14.	15.
1. 10-year CVD risk	--														
2. Neighborhood	-.19^#^	--													
3. Perceived Stress	-.13*	.40^#^	--												
4. Discrimination	-.11*	.29^#^	.20^#^	--											
5. Education	-.24^#^	-.04	.02	.13*	--										
6. Acculturation	-.10	.05	.06	.01	.36^#^	--									
7. U.S. Born	.04	-.004	-.02	.01	.29^#^	.59^#^	--								
8. Systolic BP	.43^#^	.07	-.01	.04	-.14**	-.11*	-.01	--							
9. Diastolic BP	.07	.09	.01	-.02	-.003	-.06	-.06	.60^#^	--						
10. Cholesterol	.03	.11*	-.02	.04	.05	.07	.07	.09	.18**	--					
11. HDL	-.27^#^	.04	-.10	-.13*	.09	.07	.09	-.10	-.10*	.08	--				
12. HbA1c%	.25^#^	.08	-.02	.001	-.09	.02	.02	.12*	.06	.19^#^	-.15**	--			
13. Diabetic	.40^#^	.01	.02	.08	-.09	-.10	-.07	.13*	.06	.07	-.25^#^	.58^#^	--		
14. Treated BP	-.22^#^	-.05	.06	-.06	.16**	-.03	-.13*	-.33^#^	-.18^#^	-.02	.03	-.20^#^	-.21^#^	--	
15. Smoker	-.11*	.10	.03	.11*	-.02	.05	.09	-.02	-.04	.06	-.16**	-.02	.07	-.11*	--

Note. BP = blood pressure mm/Hg, HDL = high-density lipid cholesterol mg/dL, HbA1c% = hemoglobin A1C percentage.

* *p* < .05,

** *p* < .01,

^#^ *p* < .001.

Table 3 shows the results of the hierarchical multiple regression predicting 10-year CVD risk. As the table shows, at step 1, among the systemic stressors, higher perceived neighborhood stress was predictive of lower 10-year CVD risk beyond discrimination. However, the significant effect of neighborhood stress on 10-year CVD risk disappeared when perceived stress was entered into the model. Although the effects of psychosocial stress were not moderated by acculturation or education, or their interactions, higher perceived stress remained a significant predictor of lower 10-year CVD risk. This effect remained even after adjusting for nativity in the final step.

**Table 3:** Hierarchical main and interaction effects of psychological stress, acculturation, and education on 10-year CVD risk (*n* = 391).

			10-year CVD risk		
	*R* ^*2*^	*F*	*B*	95% CI [LL, UL]	*p*
***Step 1 Systemic stressors***	.04	5.39			
Neighborhood			-.18	[-4.43, -.79]	.005
Discrimination			-.06	[-2.78,.92]	.322
***Step 2 General stressor***	.07	6.11			
Neighborhood			-.11	[-3.54,.35]	.108
Discrimination			-.04	[-2.40, 1.30]	.557
Perceived Stress			-.18	[-4.61, -.72]	.007
***Step 3 Sociocultural moderators***	.12	7.22			
Neighborhood			-.10	[-3.33,.45]	.135
Discrimination			.01	[-1.75, 1.90]	.934
Perceived Stress			-.22	[-5.08, -1.25]	.001
Acculturation			-.004	[-2.49, 2.34]	.950
Education			-.24	[-1.54, -.48]	< .001
***Step 4 Two-way interaction effects***	.14	3.34			
Neighborhood			-.10	[-3.47,.50]	.141
Discrimination			-.001	[-1.89, 1.86]	.987
Perceived Stress			-.21	[-5.01, -1.04]	.003
Acculturation			-.01	[-2.68, 2.27]	.869
Education			-.24	[-1.53, -.43]	.001
Neighborhood X Acculturation			-.003	[-2.94, 2.83]	.968
Discrimination X Acculturation			-.08	[-4.35, 1.01]	.221
Perceived Stress X Acculturation			-.02	[-2.99, 2.36]	.817
Neighborhood X Education			-.06	[-.80,.36]	.452
Discrimination X Education			-.01	[-.63,.59]	.944
Perceived Stress X Education			.09	[-.24,.99]	.235
Acculturation X Education			-.01	[-.70,.58]	.857
***Step 6 Three-way interaction effects*, *adjusted*** ^**a**^	.16	2.99			
Neighborhood			-.11	[-3.71,.44]	.122
Discrimination			-.04	[-2.56,.149]	.601
Perceived Stress			-.21	[-5.20, -.93]	.005
Acculturation			-.10	[-4.99, 1.09]	.207
Education			-.25	[-1.58, -.48]	< .001
Neighborhood X Acculturation			-.02	[-3.41, 2.60]	.790
Discrimination X Acculturation			-.09	[-4.57,.90]	.188
Perceived Stress X Acculturation			-.03	[-3.13, 2.22]	.738
Neighborhood X Education			-.04	[-.74,.43]	.601
Discrimination X Education			.03	[-.52,.79]	.682
Perceived Stress X Education			.07	[-.30,.93]	.315
Acculturation X Education			.01	[-.61,.74]	.850
Neighborhood X Acculturation X Education			.01	[-.71,.78]	.925
Discrimination X Acculturation X Education			.11	[-.20, 1.33]	.147
Perceived Stress X Acculturation X Education			.08	[-.35, 1.05]	.330
U.S. Born			.13	[-.82, 10.01]	.096

Contrary to prior work suggesting that higher levels of stress predict higher cardiovascular related risk [5–7, 9, 23], the present study showed that three forms of psychosocial stress—neighborhood stress, perceived discrimination, and perceived stress—correlated with lower 10-year CVD risk in a sample of U.S. Latino adults. Furthermore, perceived stress, above and beyond the other types of psychosocial stress, predicted lower risk. To examine whether sociocultural resources moderated these relationships, we explored the interaction between each type of psychosocial stress and acculturation and education. Our findings suggested that the interaction of acculturation and education did not moderate the effects of any of the psychosocial stresses on 10-year CVD risk.

While initially surprising, we speculate that the inverse correlation between psychosocial stress and CVD risk might be explained by limitations of coping responses within the biopsychosocial stress model. According to the biopsychosocial stress model, some people, as a coping response to stress, may not report perceiving any stress [3]. Rather they may inhibit their expression of a psychological response to stress [74]; however, they will still exhibit cardiovascular physiological responses to the stressor [75, 76]. Although these findings have been demonstrated mainly in non-Latino populations [76, 77], Ruiz and colleagues [74, 78] suggest that Latino adults may experience culturally-facilitated stress processes that may alter the perception or even appraisal of certain stimuli as stressful. Relatedly, this pattern of behavior (i.e., inhibiting expression or perception of stress) may emerge as a response to perceived available social support [74]. Indeed, past research shows that socially disadvantaged group members may be more likely to downplay psychosocial stressors, such as discrimination, when they felt they had less social support [79, 80]. However, they were less likely to minimize those stressors when they perceived social support was available. Research suggests that downplaying, denying, or ignoring stressors is a maladaptive coping mechanism that can ironically increase the negative consequences of stress [81]. Indeed, research suggests that denial of stressful events might prevent people from making important healthcare decisions or accessing medical treatment or healthcare [82].

On the other hand, participants reporting higher levels of psychosocial stress may be relying on adaptive coping resources. Research suggests that, despite their minority status in the U.S. and the enduring socioeconomic disadvantages typically associated with greater health risk, Latinos actually enjoy many health advantages compared to other groups due to sociocultural factors like their orientation toward the collective [45, 78, 83–85]. One such sociocultural factor is the tendency for Latinos to diffuse stress from the self to the collective social network, thereby allowing the collective (e.g., immediate community) to help cope with the stress, rather than feeling personally burdened [86]. This social diffusion of stress as a coping mechanism may be especially prevalent in communities that have a high concentration of similar ethnic groups, known as ethnic enclave neighborhoods [85]. Eschbach and colleagues argue that Latino adults who live in ethnic enclave neighborhoods may experience health advantages (‘barrio advantage’) due to the stronger and readily available support that is present in their communities. Participants in our study live in such an ethnic enclave neighborhood; thus, participants who reported higher levels of stress may be more accurately portraying their stressful experiences, and may have turned to their social networks within their ethnic enclave neighborhood to collectively handle the stress, thereby reducing their risk for CVD.

Interestingly, perceived stress, above and beyond the other types of psychosocial stress, predicted lower 10-year CVD risk. We interpret this finding within the context of the stress domain hypothesis, which acknowledges that how a person responds to psychosocial stress is connected to the meaning of that particular type of stress to that person [87]. The meaning of that type of stressor may be particularly contingent on sociocultural experiences [88]. For ethnic minority populations, stressors are often categorized as chronic or acute—with chronic stressors originating from systemic disparities and acute stressors representing general life events (e.g., divorce or job loss) [35, 89]. However, chronic stressors often surface and roll into general stress for ethnic minority populations. For example, for a Latino person, loss of employment, which is categorized as a general life event, may be inextricably connected to ethnic discrimination, which is a systemic or chronic stressor. Thus, perceived or general stress may be more relevant, as it comprises the systemic stress experiences, when considering health.

A second unexpected finding was that acculturation and education did not moderate the effects of psychosocial stress on 10-year CVD risk. We speculate that there are two reasons why this might have occurred. First, most of the participants did not have a high school degree and 80% had a high school degree or less (i.e., ≤ 12 years of education). Thus, we were underpowered to detect nuanced effects of education on 10-year CVD risk. Second, although we attempted to measure acculturation in a robust and multidimensional way by including measures of English-language use, childhood interaction, and preservation of culture, we did not have measures of other cultural values that may be more relevant to health. For example, cultural values that focus on family (*familismo*) and harmony (*simpatia*) facilitate more social integration, which has been linked to coping resources and is a moderator of health [14, 78, 90, 91]. Moreover, these cultural values may be more connected to social diffusion of coping, a seemingly essential component to Latino health [86].

At least three factors limit the potential generalizability of the present study. First, the stressors experienced by this community from the refinery explosion in 2005 may have affected physiological data. Unfortunately, due to the archival nature of the dataset, we were not able to ascertain timestamps within the data to identify participants who had their data collected prior to the explosion versus those who had their data collected after the explosion. Having those data would have allowed us to incorporate the role of environmental stress. Environmental chronic and acute exposure to stress can have cascading effects on biological systems by increasing blood pressure, increasing cortisol, and a host of other adverse physiological changes [60, 92, 93]. As such, the participants in this study, all of whom lived within proximity of the explosion, may have experienced adverse cardiovascular physiological changes. Thus, future research is needed to examine whether the present effects generalize to other high- and low-environmental stress Latino-dense communities.

Second, the cross-sectional nature of the data limits our understanding of how psychosocial stressors might causally influence CVD risk over time. Although we calculated likelihood of having a CVD in the next 10-years, we were unable to ascertain how risk as a function of psychosocial stress may have changed or presented for the participants after this study took place. Future research should assess CVD risk as a function of psychosocial stress over time.

Third, although we used highest level of education as an objective representation of SES, we only obtained this measure for the individual rather than for the household. Recommendations regarding objective measurements of socioeconomic position and class for health research suggest that socioeconomic data should be measured both at the individual and household level, especially among immigrants or people living in ethnic enclaves [94]. Household highest level of education may differ based on how many adults live in that house and if that level of education relates to being a main source of income for the household. Future research should incorporate highest level of household education in relation to individual health outcomes.

Despite the study’s limitations, it had at least three major strengths. First, we took advantage of data from a Latino-dense community experiencing ongoing psychosocial stress. As such, we were able to examine how sociocultural resources interact with those stressors to predict an understudied disease among Latino adults. Second, we calculated a comprehensive 10-year CVD risk using the ASCVD calculator among Latinos, as a range of biomarkers related to CVD were available. To our understanding, this is the first study to do so. Finally, our measure of acculturation was multidimensional and included several robust indicators of explicit behaviors (i.e., English-language use, childhood interactions, and preservation of culture). Prior studies measured acculturation with only time in the U.S. and/or English language acquisition [95–98].

## Conclusions

In conclusion, the present study suggests that ongoing general and systemic psychosocial stress may be linked to lower prevalence of future CVD among middle-aged and older adults of Mexican origin living in an ethnic enclave. We interpret our findings in the wider context of coping responses within the biopsychosocial stress model. We further contextualize our findings within the reserve capacity model which highlights the paradoxical health advantage that Latinos in the U.S. may have despite various and multiple socioeconomic disadvantages. Through the adoption of both a biopsychosocial framework layered with a Latino health-specific framework, we consider a more nuanced understanding of cardiovascular health and stress among Latinos.
